# Supplementary figures and images for: Improving decolorization of dyes by laccase from Bacillus licheniformis by random and site-directed mutagenesis
Source: PeerJ. 2020 Nov 11;8:e10267. doi: 10.7717/peerj.10267 (PMC7666548; doi:10.7717/peerj.10267)

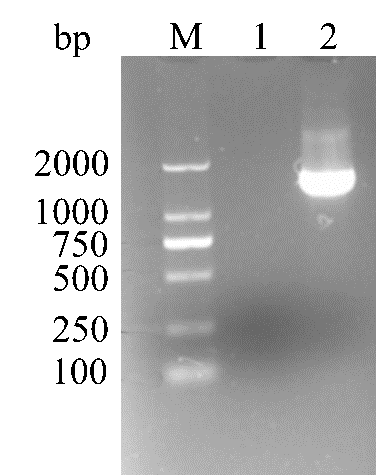

Supplement: Supplemental Information 1 — Images of strips stained with Coomassie Brilliant Blue R250. M: D2000; Lane 1: negative control; Lane 2: Cloning of Lac [file peerj-08-10267-s001.png]
